# Hypertrophy of the Heart and Effusion within the Pericardium

**Published:** 1879-07

**Authors:** Chester Hard

**Affiliations:** Ottawa, Ills.


### ARTICLE VII.

### *Hypertrophy of the Heart and Effusion within the Pericardium.*

Since his return from a visit to Germany last August, Prof. C. W. Denhood, our late county recorder, has complained of frequent attacks of difficult breathing and constriction of the chest, and at times almost amounting to suffocation, the dyspnœa becoming so great that upon more than one occasion his attendants as well as himself thought he was dying. These attacks generally came on in the night, from one to two hours after midnight, and would last until 3 or 4 o'clock, when they would pass off with cough and expectoration of glossy mucus. In the morning, he would generally go about his business, and perhaps remain free for a week or two, when, after some slight exposure to cold, he would have a recurrence of the paroxysm, which was always accompanied with great reduction of temperature, small, feeble pulse and clammy skin, from deficient oxygenation. He generally found relief from nauseants and sedatives, and sometimes chloroform and ether; these remedies he kept by him, and was in the habit of using as occasion required. About 2 o'clock on the morning of the 24th of February last, I was called to see him in what was supposed to be one of these paroxysms of asthma. I found him in a semi-recumbent posture, and breathing with great difficulty, with a wheezing or whistling noise. Pulse small and intermittent, 56 per minute; temperature, 80; skin cold and moist. He was hardly able to speak, but said that he suffered great pain from the right hip to the ankle, along the course of the sciatic nerve, but he was totally unable to move the right leg or foot. Restoratives were administered, but he rapidly sank, and died in an hour or two after I saw him, dropping away suddenly, like one falling to sleep. Eight hours after death, in company with four other physicians, I made a post-mortem examination. We found the lungs apparently healthy, but compressed and pushed out of place by the great size of the heart and its appendages, which occupied nearly thrice its ordinary space within the thorax. Not only was the heart greatly enlarged, as will be seen from the figures below, but the pericardium was enormously distended with a clear, watery fluid. The heart lay nearly transverse, and the pericardium was distended to its utmost, and looked like a large, distended bladder. After removal of the heart, the pericardium was punctured, and 1,344 C. C. of water drawn off and weighed. The auricles and ventricles were found filled with coagulated blood and fibrinous clots. After these were removed, together with the pericardium, the heart weighed 768 grams. The walls on both sides were thickened, and the whole organ greatly enlarged, but a careful examination revealed no structural disease of the valves. The question arises whether, if the true nature of the disease had been known, relief might not have been obtained by puncturing the heart case and drawing off the fluid with an aspirator; and again, would not this relief have been only temporary, and have to be repeated at intervals. He had only felt the disease troubling him since his trip to Germany some five or six months previous, but upon enquiring of his family, I learn that for four years or more he has suffered from a cough and sore throat and from slight attack of difficult breathing, but he would not call himself sick. He was a fleshy, robust looking man, of fine personal appearance, a generous liver and an indefatigable worker, being at the same time a musical professor, an editor of a weekly paper, an active and prominent politician and county recorder.

CHESTER HARD, M.D.

OTTAWA, ILLS.

